# Author Correction: KCC1 Activation protects Mice from the Development of Experimental Cerebral Malaria

**DOI:** 10.1038/s41598-020-59357-w

**Published:** 2020-02-05

**Authors:** Elinor Hortle, Lora Starrs, Fiona C. Brown, Stephen M. Jane, David J. Curtis, Brendan J. McMorran, Simon J. Foote, Gaetan Burgio

**Affiliations:** 1 Department of Immunology and Infectious Disease, John Curtin School of Medical Research, Australian National University, Australian Capital Territory, Australia; 2 Australian Centre for Blood Diseases, Central Clinical School, Monash University, Melbourne, Australia; 3 The Alfred Hospital, Melbourne, Australia; 4 Department of Medicine, Central Clinical School, Monash University, Melbourne, Australia

Correction to: *Scientific Reports* 10.1038/s41598-019-42782-x, published online 23 April 2019

The original version of this Article contained errors in the spelling of the authors Fiona C. Brown, Stephen M. Jane and David J. Curtis which were incorrectly given as Fiona Brown, Stephen Jane and David Curtis respectively.

These errors have now been corrected in the PDF and HTML versions of the Article, and in the accompanying Supplementary Information file.

