# Supplementary material for: KCC1 Activation protects Mice from the Development of Experimental Cerebral Malaria
Source: Sci Rep. 2019 Apr 23;9:6356. doi: 10.1038/s41598-019-42782-x (PMC6478876; doi:10.1038/s41598-019-42782-x)
Supplement: Supplementary file 1 — Supplementary Figures [file 41598_2019_42782_MOESM1_ESM.pdf]

Supplementary material for

KCC1 Activation protects Mice from the Development of Experimental Cerebral Malaria.

Elinor Hortle[1*], Lora Starrs[1*], Fiona C. Brown[2], Stephen M. Jane[2,4,5], David J. Curtis[2,4], Brendan J. McMorran[1], Simon J. Foote[1], and Gaetan Burgio[1]

Figure S1

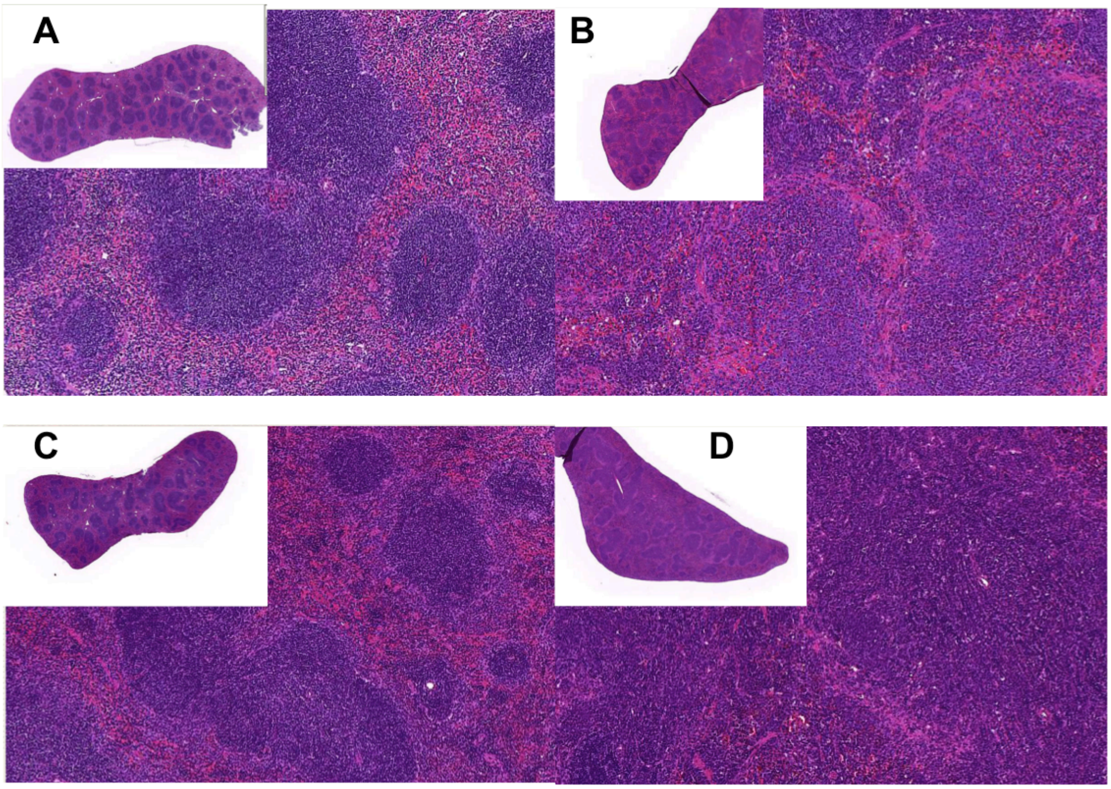

Figure S1: The M935K mutation causes an abnormal WBC response to infection. Representative H&E stained spleen sections from **(A)** uninfected WT, **(B)** infected WT, **(C)** uninfected Kcc1$^{M935K/M935K}$, and **(D)** infected Kcc1$^{M935K/M935K}$ mice. Sections are at 5x magnification; insets at 0.625x magnification.

Figure S2

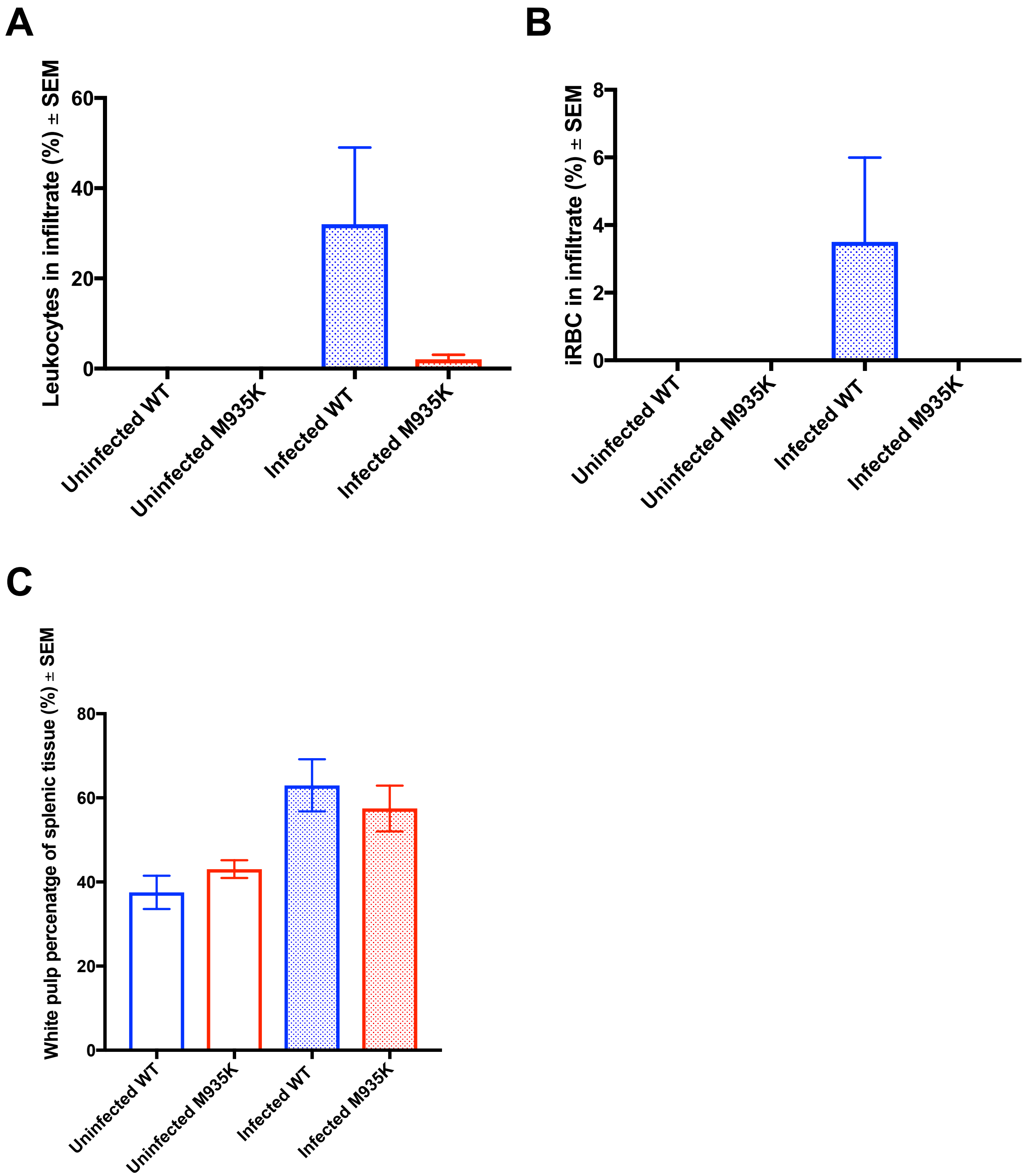
A
Leukocytes in infiltrate (%) ± SEM
60
40
20
0
Uninfected WT
Uninfected M935K
Infected WT
Infected M935K
B
iRBC in infiltrate (%) ± SEM
8
6
4
2
0
Uninfected WT
Uninfected M935K
Infected WT
Infected M935K
C
White pulp percenatge of splenic tissue (%) ± SEM
80
60
40
20
0
Uninfected WT
Uninfected M935K
Infected WT
Infected M935K

Figure S2: There is no difference in percentage of infiltrating leukocytes or infected RBCs between Kcc1$^{M935K/M935K}$ and WT mice in brain or spleen. Representative H&E stained spleen (S1) and brain sections,(Figure 3) were quantified for **(A)** average ± SEM leukocyte infiltration in the brain for uninfected and infected WT and Kcc1$^{M935K/M935K}$ mice **(B)** average ± SEM infected RBC (iRBC) in infiltrate in the brain of uninfected and infected WT and Kcc1$^{M935K/M935K}$ mice, and **(C)** average ± SEM white pulp as a percentage of total splenic tissue for uninfected and infected WT and Kcc1$^{M935K/M935K}$ mice.

Figure S3

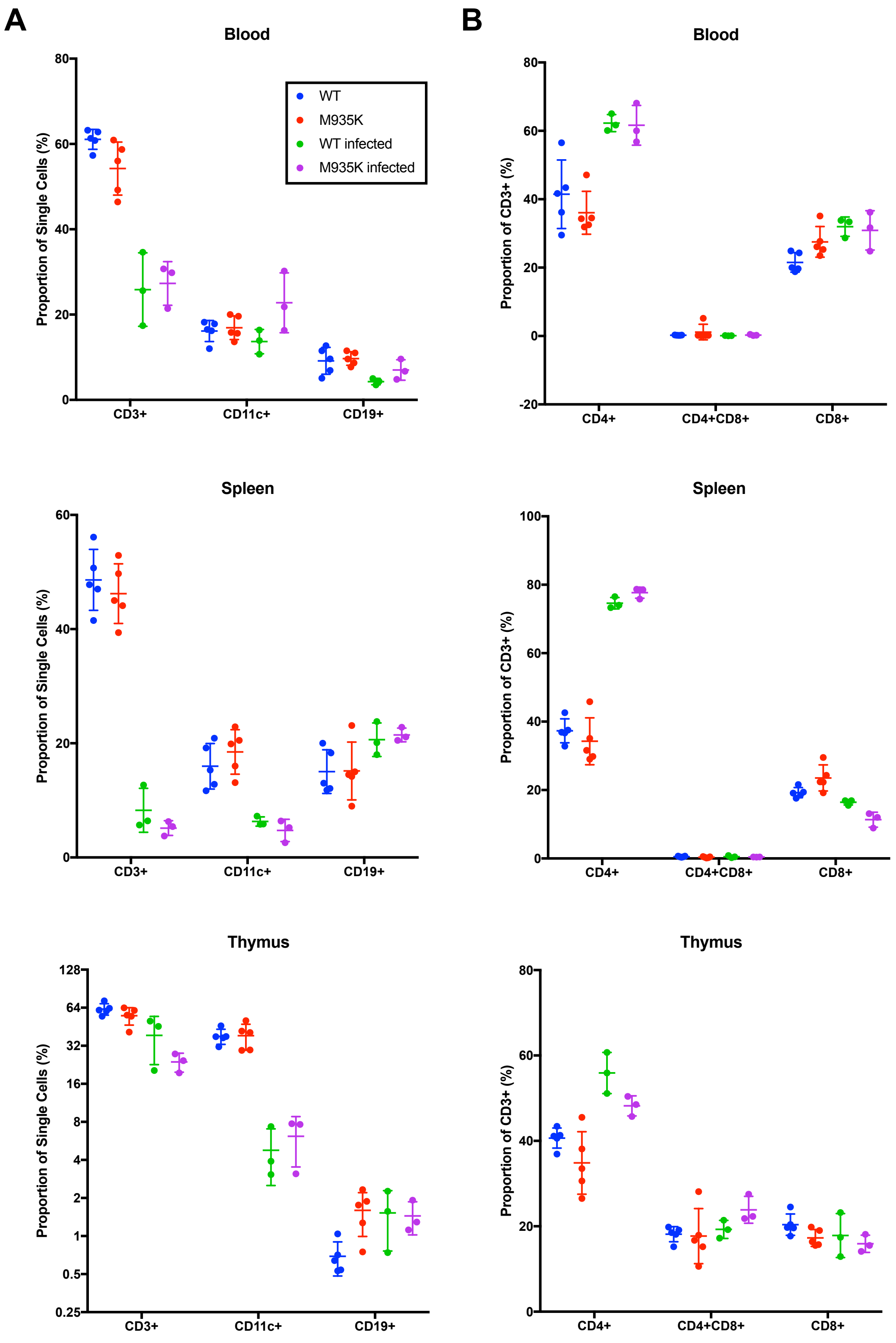

A
Blood
Proportion of Single Cells (%)
80
60
40
20
0
WT
M935K
WT infected
M935K infected
CD3+
CD11c+
CD19+
Spleen
Proportion of Single Cells (%)
60
40
20
0
CD3+
CD11c+
CD19+
Thymus
Proportion of Single Cells (%)
128
64
32
16
8
4
2
1
0.5
0.25
CD3+
CD11c+
CD19+
B
Blood
Proportion of CD3+ (%)
80
60
40
20
0
-20
CD4+
CD4+CD8+
CD8+
Spleen
Proportion of CD3+ (%)
100
80
60
40
20
0
CD4+
CD4+CD8+
CD8+
Thymus
Proportion of CD3+ (%)
80
60
40
20
0
CD4+
CD4+CD8+
CD8+

Figure S3: The Kcc1^{M935K} mutation does not alter immune cell populations in the blood, spleen, or thymus. (A) Average ± SEM proportion of lymphocytes that are CD3+, CD11c+, and CD19+ in the blood spleen and thymus. **(B)** Average ± SEM proportion of CD3+ cells that are CD4+, CD8+, and CD4+CD8+ in the blood spleen and thymus. Black circles = uninfected WT (n=5), grey circles = uninfected Kcc1$^{M935K/M935K}$ (n=5), black open squared = infected WT (n=3), grey open squares = infected Kcc1$^{M935K/M935K}$ (n=3).

Figure S4

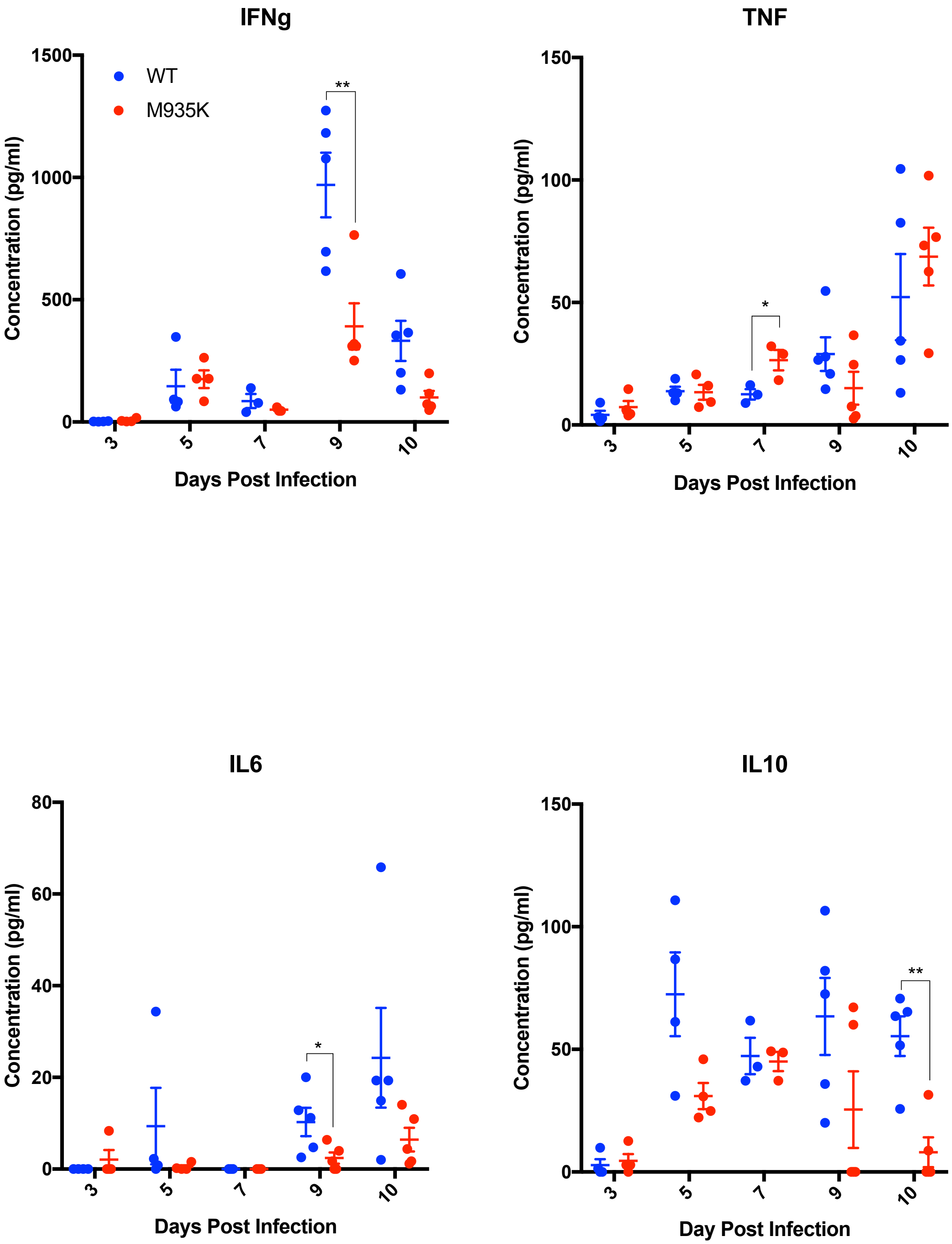

IFNg
WT
M935K
**
Concentration (pg/ml)
1500
1000
500
0
3
5
7
9
10
Days Post Infection
TNF
*
Concentration (pg/ml)
150
100
50
0
3
5
7
9
10
Days Post Infection
IL6
*
Concentration (pg/ml)
80
60
40
20
0
3
5
7
9
10
Days Post Infection
IL10
**
Concentration (pg/ml)
150
100
50
0
3
5
7
9
10
Day Post Infection

Figure S3: The Kcc1^{M935K} mutation alters the inflammatory response to *P. berghei* infection. Average ± SEM concentration of cytokines in the plasma of WT and Kcc1$^{M935K/M935K}$ mice during infection with *P. berghei*. **P<0.01, Significance calculated using unpaired t test
